# Immediate Early Genes Anchor a Biological Pathway of Proteins Required for Memory Formation, Long-Term Depression and Risk for Schizophrenia

**DOI:** 10.3389/fnbeh.2018.00023

**Published:** 2018-02-19

**Authors:** Ketan K. Marballi, Amelia L. Gallitano

**Affiliations:** Department of Basic Medical Sciences and Psychiatry, University of Arizona College of Medicine—Phoenix, Phoenix, AZ, United States

**Keywords:** schizophrenia, immediate early gene, long-term depression, EGR3, ARC, NFATC3, EGR1, Nab2

## Abstract

While the causes of myriad medical and infectious illnesses have been identified, the etiologies of neuropsychiatric illnesses remain elusive. This is due to two major obstacles. First, the risk for neuropsychiatric disorders, such as schizophrenia, is determined by both genetic and environmental factors. Second, numerous genes influence susceptibility for these illnesses. Genome-wide association studies have identified at least 108 genomic loci for schizophrenia, and more are expected to be published shortly. In addition, numerous biological processes contribute to the neuropathology underlying schizophrenia. These include immune dysfunction, synaptic and myelination deficits, vascular abnormalities, growth factor disruption, and N-methyl-D-aspartate receptor (NMDAR) hypofunction. However, the field of psychiatric genetics lacks a unifying model to explain how environment may interact with numerous genes to influence these various biological processes and cause schizophrenia. Here we describe a biological cascade of proteins that are activated in response to environmental stimuli such as stress, a schizophrenia risk factor. The central proteins in this pathway are critical mediators of memory formation and a particular form of hippocampal synaptic plasticity, long-term depression (LTD). Each of these proteins is also implicated in schizophrenia risk. In fact, the pathway includes four genes that map to the 108 loci associated with schizophrenia: *GRIN2A*, nuclear factor of activated T-cells (*NFATc3*), early growth response 1 (*EGR1*) and NGFI-A Binding Protein 2 (*NAB2*); each of which contains the “Index single nucleotide polymorphism (SNP)” (most SNP) at its respective locus. Environmental stimuli activate this biological pathway in neurons, resulting in induction of *EGR* immediate early genes: *EGR1*, *EGR3* and *NAB2*. We hypothesize that dysfunction in any of the genes in this pathway disrupts the normal activation of *Egr*s in response to stress. This may result in insufficient electrophysiologic, immunologic, and neuroprotective, processes that these genes normally mediate. Continued adverse environmental experiences, over time, may thereby result in neuropathology that gives rise to the symptoms of schizophrenia. By combining multiple genes associated with schizophrenia susceptibility, in a functional cascade triggered by neuronal activity, the proposed biological pathway provides an explanation for both the polygenic and environmental influences that determine the complex etiology of this mental illness.

## Introduction

Since the start of the 21st century, tremendous advances have been made in the identification of genes associated with risk for major neuropsychiatric illnesses such as schizophrenia, bipolar disorder and depression. However, the field of psychiatry still lags behind other areas of medicine in identifying even a single gene that has been definitively demonstrated to cause any of these mental illnesses. This is due in large part to the complex genetics that underlie these disorders. Now, 15 years after the landmark publication that identified Neuregulin 1 (NRG1) as a candidate gene for schizophrenia (Stefansson et al., 2002), there remain two unanswered questions at the forefront of the field of psychiatric genetics: (1) how can the polygenic nature of susceptibility to schizophrenia be explained? and (2) how do genes implicated in risk for schizophrenia interact with environmental factors to give rise to the disorder?

To address both of these questions, we have proposed the hypothesis that numerous schizophrenia susceptibility genes form a critical signaling pathway that is responsive to environmental stress. Dysfunction of any of the proteins in this pathway would reduce the normal activation of a key transcription factor, early growth response 3 (*EGR3*), an immediate early gene that is both associated with schizophrenia in humans (Yamada et al., 2007; Kim et al., 2010; Ning et al., 2012; Zhang et al., 2012; Huentelman et al., 2015), and expressed at reduced levels in patients’ brains (Mexal et al., 2005; Yamada et al., 2007). As a critical mediator of numerous biological processes disrupted in schizophrenia, such as growth factor signaling, myelination, vascularization, immune function, memory formation and synaptic plasticity (Gallitano-Mendel et al., 2007; Jones et al., 2007; Suehiro et al., 2010; Li et al., 2012; Kurosaka et al., 2017), insufficient activation of *EGR3* may result in neuropathology that gives rise to schizophrenia.

Schizophrenia risk is influenced by many genes in addition to environmental factors. The illness has a prevalence rate of roughly 1% worldwide, and its cause remains unknown. Studies show concordance rates of approximately 50% in monozygotic twins, roughly twice that of dizygotic twins, indicating that there are both genetic and non-genetic determinants of schizophrenia (McGue and Gottesman, 1991). Stressful events are associated with schizophrenia risk. These include prenatal stress such as nutritional deficiency, or exposure to famine, infection (e.g., rubella, influenza, *Toxoplasma gondii* and herpes simplex virus), or maternal stress. Stress during the perinatal period and early life also increase risk for the illness. Examples include obstetric complications and perinatal trauma, and stressful life events such as childhood trauma (Corcoran et al., 2001, 2003; Mittal et al., 2008; van Winkel et al., 2008; Brown and Derkits, 2010; Brown, 2011). Adding to the complex etiology of this illness, the most recent genome-wide association study (GWAS) of single nucleotide polymorphisms (SNPs) identified 108 genomic loci that influence schizophrenia susceptibility (Schizophrenia Working Group of the Psychiatric Genomics Consortium, 2014). To date, there is no consensus on a mechanism to explain how so many genetic variations interact with environmental factors to cause schizophrenia.

## Identifying A Pathway

Immediate early genes are a class of genes that are rapidly induced in response to a stimulus, in a manner that is independent of protein synthesis. In the brain, they are expressed within minutes of neuronal activity triggered by environmental stimuli. A large number of immediate early genes encode proteins that function as transcription factors (termed immediate early gene transcription factors (Curran and Morgan, 1995)). These genes are thus poised to translate changes in the environment into long-term changes in the brain through the regulation of their target genes. This presumably underlies the critical role of many immediate early gene transcription factors in memory formation, a process that requires long-term encoding of environmental experiences.

We have hypothesized that this function of immediate early gene transcription factors, as key regulators of the brain’s gene-expression response to experience, uniquely positions them to mediate the dual genetic and environmental influences on schizophrenia susceptibility (Gallitano-Mendel et al., 2008). We focus on the *Egr* family of immediate early genes since they are activated in response to changes in the environment (Senba and Ueyama, 1997; Martinez et al., 2002), and regulate fundamental processes in the nervous system that are known to be dysfunctional in schizophrenia. These include myelination, vascularization, learning and memory, and synaptic plasticity (Paulsen et al., 1995; Guzowski et al., 2001; Nagarajan et al., 2001; Bozon et al., 2002, 2003; Flynn et al., 2003; Crabtree and Gogos, 2014). In addition, *Egrs* are activated downstream of N-methyl-D-aspartate receptors (NMDARs; Cole et al., 1989) and growth factors (Schulze et al., 2008; Shin et al., 2010), dysfunction of which have each been hypothesized to contribute to schizophrenia susceptibility (Olney et al., 1999; Moises et al., 2002; Calabrese et al., 2016).

We hypothesize that variations that reduce the normal amount of *Egr* gene expression in response to environmental stimuli would result in lower than normal levels function of these processes. Specifically, this would result in insufficient activation of *Egr* target genes, such as brain-derived neurotrophic factor (BDNF) and activity-regulated cytoskeleton associated protein (*Arc*/*Arg3*.*1*: hereafter referred to as “*Arc*”; Li et al., 2005; Maple et al., 2017; Meyers et al., 2017a,b). Lower than normal activation of these genes could account for the reduced levels of white matter, reduced synaptic spine density, and deficiencies in memory and cognitive processing, that are seen in patients with schizophrenia, or hypothesized to underlie its pathogenesis (Ingvar and Franzen, 1974; Saykin et al., 1991; Glantz and Lewis, 2000; Moises et al., 2002; Davis et al., 2003).

Several findings led us to focus specifically on *Egr* family member *Egr3* as we investigated this hypothesis. First, *NRG1* was identified as a schizophrenia candidate gene in a large-scale genetic association study (Stefansson et al., 2002). In mice, *Nrg1* was found to be essential to maintain *Egr3* expression in the peripheral muscle spindle (Hippenmeyer et al., 2002). Subsequently the protein phosphatase calcineurin (CN) was identified as a schizophrenia candidate protein based on the phenotype of *CN*^−/−^ mice, and the association of the gene encoding one of its subunits (*PPP3CC*) with schizophrenia in a Japanese population (Gerber et al., 2003). CN had also been shown to regulate expression of *Egr3* in the immune system (Mittelstadt and Ashwell, 1998). Together, these findings indicated that *Egr3* was regulated downstream of three proteins independently implicated in schizophrenia risk: NMDARs, NRG1 and CN.

To answer whether *Egr3* may play a role in schizophrenia, we investigated the behavior and physiology of *Egr3*-deficient (^−/−^) mice. Our studies revealed that *Egr3*^−/−^ mice display behavioral abnormalities consistent with animal models of schizophrenia. These include locomotor hyperactivity, a behavior produced in rodents treated with drugs that cause psychosis in humans, such as NMDAR antagonists—phencyclidine and ketamine, and dopaminergic agents including amphetamines (Lipska and Weinberger, 2000; Tamminga et al., 2003). The fact that this activity is reversible with antipsychotic medications further validates this as a phenotype relevant to schizophrenia (Freed et al., 1984; O’Neill and Shaw, 1999). *Egr3*^−/−^ mice show locomotor hyperactivity in response to a novel environment that is reversed with antipsychotic treatment (Gallitano-Mendel et al., 2008). Schizophrenia is also characterized by cognitive deficits, which a feature that led to Emil Kraepelin to define the syndrome “Dementia Praecox” (Kraepelin and Robertson, 1919). *Egr3*^−/−^ mice have deficits in spatial memory as well as defects in hippocampal long-term depression (LTD), a form of synaptic plasticity (Gallitano-Mendel et al., 2007, 2008) normally activated in response to stress and novelty (Manahan-Vaughan and Braunewell, 1999; Kemp and Manahan-Vaughan, 2007). Notably, both NMDARs and CN, proteins that regulate induction of *Egr3*, are also required for memory formation and LTD. This suggested that EGR3 was functioning in a signal transduction cascade of proteins that were activated in the brain in response to novelty and stress, and required for both memory formation and LTD (Gallitano-Mendel et al., 2007).

We subsequently described that numerous of the key proteins in this pathway were associated with risk for schizophrenia (Gallitano-Mendel et al., 2008). This led us to hypothesize that other genes in this pathway that shared the characteristics of regulating memory and hippocampal LTD, such as the EGR3 target gene *ARC*, should also be candidates for a role in schizophrenia susceptibility (Gallitano, 2008). Indeed, numerous genome wide association studies of copy number variations (CNVs), *de novo* mutations and SNPs (Kirov et al., 2012; Fromer et al., 2014; Purcell et al., 2014), as well as our own resequencing study that identified a schizophrenia associated *ARC* SNP (Huentelman et al., 2015), have subsequently supported this hypothesis.

**Figure 1:**
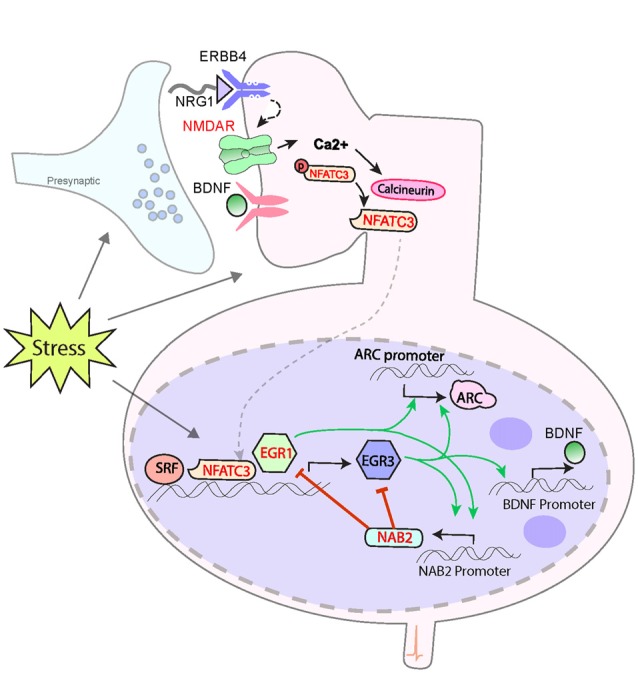
Model for a biological pathway regulating memory, synaptic plasticity and schizophrenia risk. Numerous schizophrenia-risk associated proteins act either upstream or downstream of immediate early genes *EGR3* and *ARC*. Red labels indicate proteins encoded by genes that map to the 108 loci for schizophrenia risk. Both EGR3 and ARC are activated by environmental events, such as stress, via activity-dependent activation of NMDARs. The resulting influx of Ca++ triggers Calcineurin/(CN) nuclear factors of activated T-cell (NFAT) signaling which can induce *EGR3* expression. EGR3 positively regulates, and maintains expression of, *ARC*. NGFI-A Binding Protein 2 (NAB2), which is a transcriptional target of EGR3 and EGR1, binds to EGR proteins (EGR1, 2 and 3) to co-regulate expression of target genes. This action includes feedback inhibition of EGR1 and EGR3, and NAB2 itself, a critical element of the temporal regulation of activity-dependent genes. Additional proteins that interact in the pathway include NRG1 which, via binding to ERBB receptors, maintains expression of EGR3 (in muscle spindles), and has been shown to activate NMDARs. BDNF, via binding to Trk-B receptors, regulates EGR3-mediated expression of NMDAR subunit NR1. EGR3, in turn, is required for activity-dependent expression of BDNF. SRF is a transcription factor required for memory formation and long-term depression (LTD), which regulates expression of EGR3 (and EGR1). Perturbations in any components of this biological pathway are expected to result in deficient induction of EGR3, and its target genes, in response to neuronal activity, including that triggered by stress. Insufficient activation of this pathway in response to stimuli may result in poor memory formation and contribute to cognitive deficits. Deficient activation of the pathway in response to stress may also result in insufficient neuroprotective processes and thereby contribute, over time, to the neuropathology that gives rise to schizophrenia (see Figure 2). Note that many interactions represented are taken from literature on studies in immune system and have yet to be validated in the brain. The shared roles of these proteins in the immune system may contribute to association between schizophrenia and immune dysfunction. Abbreviations: NMDAR, N-methyl d-aspartate receptor; NFATC3, nuclear factor of activated T-cells; EGR1, 3, Early growth response 1, 3; ARC, Activity-regulated cytoskeleton-associated protein. Additional potential contributing proteins are indicated in gray include: SRF, serum response factor; BDNF, brain-derived neurotrophic factor; NRG1, neuregulin 1; ErbB4, ErbB2 receptor tyrosine kinase 4, Trk-B neurotrophic receptor tyrosine kinase 2. References: Yamagata et al. (1994); Senba and Ueyama (1997); Mittelstadt and Ashwell (1998); Rengarajan et al. (2000); Hao et al. (2003); Jacobson et al. (2004); Kumbrink et al. (2005, 2010); Li et al. (2005); Ramanan et al. (2005); Etkin et al. (2006); Lindecke et al. (2006); Bjarnadottir et al. (2007); Gallitano-Mendel et al. (2007, 2008); Gallitano (2008); Marrone et al. (2012); Herndon et al. (2013); Ramirez-Amaya et al. (2013); Schizophrenia Working Group of the Psychiatric Genomics Consortium (2014); Maple et al. (2015); Pouget et al. (2016); Meyers et al. (2017a,b); Ramanan (personal communication).

Here we present the key proteins that comprise our proposed biological pathway, shown in Figure 1. We begin with a brief review of hippocampal LTD, highlighting its response to stress. We follow with a section devoted to each protein in the pathway. For each protein we: (1) summarize the evidence demonstrating the regulatory relationships that support its position in the pathway; (2) outline the findings indicating its roles in processes that are disrupted in schizophrenia; (3) mention supporting studies that demonstrate its genetic association with risk for the illness (summarized in Supplementary Table S1); and (4) if known, altered levels in the brains of patients with schizophrenia. When available, we will also review the role for each in synaptic plasticity, focusing on LTD, and define the relationship of the protein being discussed with EGR3. We will include a brief section on additional proteins for which there is new evidence indicating a potential contribution to this pathway. We briefly review the role of LTD in the neurobiological response to stress and novelty. In the Discussion we propose our hypothesis that this biological pathway mediates protective neurobiological responses to stress. We then present a model to explain how genetic variations in genes of this pathway may produce a predisposition to schizophrenia that results in illness in a manner dependent upon the stress history of an individual.

## Long-Term Depression, Stress and Memory

### LTD

LTD is a form of synaptic plasticity in which stimulation results in a prolonged decrease in strength of the synaptic connection (reviewed in Malenka and Bear, 2004; Kemp and Manahan-Vaughan, 2007; Collingridge et al., 2010). One of the most well-investigated forms of LTD is that produced by low frequency stimulation of the hippocampal Schaffer Collateral pathway, the axonal projections from CA3 neurons that synapse onto CA1 pyramidal neurons. This process is essential for spatial memory consolidation, and induces expression of immediate early genes. Moreover, the stimulation that induces the highest levels of immediate early genes also results in the longest duration of LTD (Abraham et al., 1994) suggesting an important functional role for immediate early genes in this form of synaptic plasticity. The low frequency stimulation that induces LTD also requires the function of NMDARs, as well as numerous proteins in our proposed biological pathway for schizophrenia susceptibility.

Compared to its counterpart long-term potentiation (LTP), LTD has received much less investigative attention, and is thus less well understood. NMDAR activation is an essential step in establishment of low frequency stimulation-induced hippocampal LTD (Dudek and Bear, 1992; Mulkey and Malenka, 1992). Both *GluN1* and *GluN2B* subunits have also been implicated in mediating this form of LTD (Kutsuwada et al., 1996; Manahan-Vaughan and Braunewell, 1999; Liu et al., 2004). Early studies demonstrated that mice lacking the specific *GluN2B*-containing NMDAR isoform had deficits in LTD (Kutsuwada et al., 1996; Brigman et al., 2010). However, recent work investigating the role of GLUN2B in this form of synaptic plasticity have produced differing results, some of which may be explained by technical differences in study methodologies (Chen and Bear, 2007; Bartlett et al., 2011; Shipton and Paulsen, 2014).

As described in the sections below, numerous downstream effectors of NMDAR activation have also been shown to be critical for activity dependent LTD. These include CN, EGR3, ARC, serum response factor (SRF) and BDNF (Zeng et al., 2001; Etkin et al., 2006; Plath et al., 2006; Gallitano-Mendel et al., 2007; Beazely et al., 2009; Mizui et al., 2015; Novkovic et al., 2015; Bukalo et al., 2016; Kojima and Mizui, 2017). The roles of several of the proteins that comprise our proposed pathway for schizophrenia risk are not limited to this form of synaptic plasticity. For example, NMDARs, BDNF and ARC are also required for LTP. Interestingly, dysfunction in LTP has also been hypothesized to play a role in schizophrenia (Rison and Stanton, 1995). However, the shared role of numerous core pathway genes in the process of LTD stood out to us early on in the recognition of this biological cascade (Gallitano-Mendel et al., 2007, 2008). Since LTD had received so much less emphasis, at that time, this seemed a coincidence worthy of further investigation.

Several features of LTD make this form of synaptic plasticity of particular interest for a potential contribution to the development of schizophrenia. Stress is one of the major environmental risk factors for this schizophrenia (Holtzman et al., 2013). Numerous studies have demonstrated a relationship between stress and LTD (Rowan et al., 1998). For example, while stress inhibits establishment of LTP in the hippocampal CA1 region, it augments LTD (Kim et al., 1996; Xu et al., 1997; Artola et al., 2006). The association of stress with schizophrenia susceptibility suggests the possibility that disruption of the normal LTD response to stress could result in pathology that may increase risk for this, and potentially other, psychiatric illness.

A second intriguing role of LTD is that of encoding long-term plasticity in response to novelty. Studies involving numerous genes in the proposed biological pathway have demonstrated an association between acquisition and retention of novel information and establishment of LTD (Manahan-Vaughan and Braunewell, 1999; Artola et al., 2006; Etkin et al., 2006; Plath et al., 2006; Gallitano-Mendel et al., 2007; Kemp and Manahan-Vaughan, 2007). Exposure to novelty enhances LTD (Manahan-Vaughan and Braunewell, 1999) and also induces expression of pathway genes such as *Egr3* and *Arc*, the latter of which persists in the hippocampal dentate gyrus for up to 8 h (Marrone et al., 2012; Ramirez-Amaya et al., 2013).

We have recently reported that this persistent expression of *Arc* induced by a brief novel exposure requires *Egr3* (Maple et al., 2017). We have also reported that *Egr3* deficient mice, which have deficits in hippocampal LTD, also demonstrate hyper-reactivity to novel environments. Not only does exploration of a novel location induce LTD, but the reverse is also true; that low frequency stimulation of the hippocampus *in vivo* facilitates the exploratory behavior of rodents (Manahan-Vaughan and Braunewell, 1999). It is intriguing to consider whether dysfunction in these processes in humans may contribute to the cognitive and memory deficits that are a key feature of schizophrenia. These findings suggest it will be of great interest to investigate whether other genes recently identified as candidates for influencing schizophrenia risk may also play a role in this specific form of hippocampal synaptic plasticity.

### Response to Stress and Novelty

Stress and exposure to novelty are two of the major stimuli that activate expression of immediate early genes, including *EGR3* and *ARC*, key output proteins in our proposed biological pathway (Senba and Ueyama, 1997; Marrone et al., 2012; Ramirez-Amaya et al., 2013; Maple et al., 2015). These stimuli also facilitate induction of hippocampal LTD, a form of synaptic plasticity associated with the formation and retention of immediate memories of novel experiences (Rowan et al., 1998; Manahan-Vaughan and Braunewell, 1999; Xiong et al., 2003; Artola et al., 2006; Kemp and Manahan-Vaughan, 2007; Collingridge et al., 2010).

Our prior work characterizing the phenotypes of *Egr3*^−/−^ mice revealed that *Egr3* is required for the normal response to stress and novelty (Gallitano-Mendel et al., 2007). Similar behavioral abnormalities in the stress and novelty responses have been reported in preclinical studies of other genes in the proposed pathway including CN, SRF and ARC (Miyakawa et al., 2003; Etkin et al., 2006; Kozlovsky et al., 2008; Bramham et al., 2010; Weinstock, 2017).

Schizophrenia is also characterized by a heightened sensitivity to stress, abnormal sensorimotor gating in response to stressful stimuli, and abnormalities in habituation to novel and stressful stimuli (Braff et al., 1992; Corcoran et al., 2001; van Os et al., 2010; Holtzman et al., 2013; Kahn et al., 2015). The fact that LTD is facilitated by novelty and stress, and is disrupted when genes of our proposed pathway, numerous of which are associated with schizophrenia risk, are dysfunctional, suggests an intriguing link between this form of synaptic plasticity and the processes that are awry in schizophrenia.

## Proteins That Form the Pathway

### NMDA Receptor

NMDARs are activated in response to neuronal depolarization, which occurs when the brain responds to stimuli in the environment. NMDARs are one of three major classes of ligand-gated ionotropic receptors for glutamate in the brain. They are multi-subunit Ca++ channels, formed by hetero-tetramers of two glycine-binding (GLUN1), and two glutamate-binding (GLUN2), subunits (Papadia and Hardingham, 2007). The GLUN1 subunit, which has eight isoforms, is encoded by the *GRIN1* gene. In contrast, there are four subtypes of GLUN2 subunits (GLUN2A, GLUN2B, GLUN2C and GLUN2D), which are encoded by four different genes (*GRIN2A*, *GRIN2B*, *GRIN2C* and *GRIN2D*, respectively). Each subunit has three distinct domains, an external N-terminal domain, a transmembrane domain that forms the channel pore, and an internal C terminal domain (Carvajal et al., 2016). The NMDAR is activated when glutamate binds to the GLUN2 subunit, coincident with binding of co-agonists magnesium and glycine/ D-serine to GLUN1 (Balu, 2016). Depolarization of the neuron is essential for NMDAR function, as this change in charge is required to remove the Mg++ ion that blocks the channel pore region, thereby allowing calcium to enter the neuron.

#### Evidence for a Role in Schizophrenia

The NMDAR hypofunction model of schizophrenia was proposed in the 1990’s (Javitt and Zukin, 1991; Olney et al., 1999) based on studies highlighting the ability of NMDAR antagonists, phencyclidine and ketamine, to cause psychosis and memory impairments in humans. The effect of these drugs in rodents were the basis for subsequently defining “schizophrenia-like” behavioral abnormalities in animal models (Malhotra et al., 1997; Lahti et al., 2001; Amitai and Markou, 2010; van den Buuse, 2010).

Mice deficient for the *GluN1* subunit of the NMDAR show schizophrenia-like behavioral abnormalities that are reversed with antipsychotic treatment (Mohn et al., 1999). Studies employing a conditional knockout of the *GluN1*subunit have been used to identify specific cell types in which disruption of the receptor results in cognitive and behavioral defects. Selective disruption of the *GluN1* gene in excitatory neurons of layer II/III of the prefrontal and sensory cortices of mice causes impairments in short-term memory and prepulse inhibition (Rompala et al., 2013), and *GluN1* gene inactivation in parvalbumin interneurons caused deficits in habituation, working memory and associative learning (Carlén et al., 2012).

Genetic studies also support the importance of NMDAR genes in schizophrenia. Genome wide significant associations with schizophrenia have been reported for at least two of the NMDAR genes- *GRIN2A* and *GRIN2B* (Taylor et al., 2016). *GRIN2A* was recently shown to be associated with schizophrenia in the largest GWAS to date (Schizophrenia Working Group of the Psychiatric Genomics Consortium, 2014). Moreover, it is the sole gene at the locus (108 loci-region 82), and thereby also contains the “Index SNP”, defined as the SNP with the greatest significance at the disease-associated genomic locus (Please refer to Supplementary Table S1 for details of all index SNPs found in genes from our biological pathway). Genetic variation in the *GRIN2B* gene has also been reported to be associated with schizophrenia (Awadalla et al., 2010; Demontis et al., 2011; Kenny et al., 2014).

A large exome sequencing study of *de novo* mutations in schizophrenia conducted by Fromer et al. (2014) used DNA from 623 family “trios” (affected individual and both parents) to identify novel mutations in schizophrenia patients. They found that *de novo* mutations are increased in schizophrenia patients compared to the general population and, that these mutations were enriched in genes encoding proteins forming the NMDAR complex (Fromer et al., 2014), further supporting the hypothesis that dysfunction of NMDARs, or their downstream processes, increases risk for schizophrenia.

Postmortem brain tissue studies have shown region-specific alterations in NMDAR subunits at both the gene and protein levels. Weickert et al. (2013) showed decreased GRIN1 gene and protein expression, and decreased *GRIN2C* gene expression, in the dorsolateral prefrontal cortex regions of schizophrenia patients compared to controls. In a separate study, Gao et al. (2000) demonstrated high levels of *GRIN1* and low levels of *GRIN2C* mRNA in postmortem hippocampal samples from schizophrenia patients compared with healthy controls.

#### Role in LTD

NMDARs have long been recognized to play critical roles in hippocampal long-term potentiation (LTP) and LTD, forms of synaptic plasticity that correlate with learning and memory (Bear and Malenka, 1994; Malenka and Bear, 2004). LTP is defined as the activity-dependent strengthening of synapses (Scharf et al., 2002), while LTD refers to an activity-dependent decrease in synaptic strength (Mulkey et al., 1994). Although NMDARs influence both forms of synaptic plasticity, a role in LTD appears to be a feature of numerous genes functioning both upstream and downstream of EGR3, and is therefore the focus of this review.

LTD mediated by NMDARs is crucial for consolidation of hippocampal dependent memory (Wong et al., 2007; Brigman et al., 2010; Ge et al., 2010), and alterations in the ratio of GLUN2A:GLUN2B subunits affects levels of LTD induced by a low frequency (3–5 Hz) electrical stimulation (Cui et al., 2013). Mice that lack *GluN2B* in pyramidal neurons in the cortex and CA1 regions of the hippocampus show impaired NMDAR-mediated LTD, and decreased dendritic spine density in CA1, compared to wildtype mice (Brigman et al., 2010).

#### Relationship to EGR3

The connection between NMDAR activation and *Egr3* gene expression was first shown by Yamagata et al. (1994), who demonstrated that high frequency stimulation of rat hippocampal neurons that induces LTP also activates expression of *Egr3*. Both *Egr3* expression and establishment of LTP are blocked by pretreatment with the NMDAR antagonist MK-801. Thus, this form of activity-dependent expression of *Egr3* in the hippocampus requires NMDAR function.

Interestingly, *Egr3* also influences the function of NMDARs. *Egr3*^−/−^ mice display deficits in the electrophysiologic response of hippocampal neurons to drugs selective for GLUN2B-containing NMDARs (Gallitano-Mendel et al., 2007). However, the fact that quantitative reverse transcriptase polymerase chain reaction (qRT-PCR) analysis did not reveal differences in the levels of *Grin2b* mRNA in *Egr3*^−/−^ mice compared with controls, suggests that EGR3 may not be directly regulating expression of this NMDAR subunit (Gallitano-Mendel et al., 2007). Others have reported that overexpression of *Egr3* (but not *Egr1*) *in vitro* regulates expression of an NMDAR subunit *NR1*-promoter-driven reporter construct, a response that is BDNF dependent (Kim et al., 2012). Thus, *Egr3* expression is induced by neuronal activity in an NMDAR-dependent fashion. In addition, *Egr3* is also required for function of GLUN2B-containing NMDARs, though the mechanisms underlying this regulation have not yet been established.

### Calcineurin

A key process triggered by calcium influx through NMDARs is the activation of calcium-dependent phosphatases, including CN (Horne and Dell’Acqua, 2007). CN is a multi-subunit protein consisting of two main subunits: the catalytic subunit calcineurin A, and the calcium binding regulatory subunit calcineurin B (Rusnak and Mertz, 2000). CN plays important roles in both the immune system and the brain. In T-cells, mitogenic stimuli increase levels of intracellular calcium, causing calmodulin-mediated activation of CN. CN then dephosphorylates nuclear factors of activated T-cell (NFAT) in the cytoplasm, causing its translocation to the nucleus where it regulates expression of target genes (Rao et al., 1997). In the nervous system, CN acts in conjunction with NFAT proteins to regulate numerous critical processes—reviewed elegantly by Kipanyula et al. (2016). These range from developmental roles in corticogenesis (Artegiani et al., 2015) and myelination (Kao et al., 2009), to activity dependent synaptogenesis (Ulrich et al., 2012), to glial cell activation, particularly in the context of neuroinflammation (Neria et al., 2013).

#### Evidence for a Role in Schizophrenia

An increasing body of *in vivo* and genetic evidence suggests a role for CN in the pathogenesis of schizophrenia. Immunosuppressive drugs that block CN, such as cyclosporin A and FK-506 produce side effects reminiscent of the symptoms of schizophrenia. These include memory impairment, auditory and visual hallucinations, paranoia, depression, and flattened affect (Bechstein, 2000; Porteous, 2008; American Psychiatric Association, 2013). Forebrain specific CN knockout mice show cognitive and behavioral abnormalities consistent with animal models of schizophrenia, such as working memory deficits (Zeng et al., 2001), increased locomotor activity and abnormalities in attention, social interaction and nesting behavior (Miyakawa et al., 2003).

Human genetic studies have identified associations between variations in *PPP3CC*, the gene that encodes the α-1 subunit of calcineurin A, and schizophrenia, in both Caucasian (Gerber et al., 2003) and Asian populations (Horiuchi et al., 2007; Liu et al., 2007; Yamada et al., 2007). Yamada et al. (2007) noted that *EGR3* was located near *PPP3CC* (within a 252 kb interval) on the short arm of chromosome 8, and showed through linkage disequilibrium studies that they form distinct regions of schizophrenia susceptibility. Although *PPP3CC* is not listed as a candidate gene at one of the 108 loci associated with schizophrenia, numerous other non-calcineurin protein phosphatase genes do map to the loci identified in that GWAS. These include: *PPP1R13B*, *PPP1R16B*, *PPP2R3A* and *PPP4C*. This suggests the importance of protein-phosphatases in the etiology of schizophrenia. Notably serine/threonine phosphatase PP1/PP2A is a crucial player in synaptic plasticity and LTD (Mulkey et al., 1994).

#### Role in LTD

The forebrain specific CN knockout mice that display schizophrenia-like behavioral abnormalities also have disrupted hippocampal LTD, as well as deficits in hippocampal working and episodic memory (Zeng et al., 2001). Notably, this hippocampal LTD deficit phenotype is also seen in *Egr3*^−/−^ mice (Gallitano-Mendel et al., 2007). Several additional lines of evidence suggest CN to be a crucial regulator of LTD. CN regulates dephosphorylation, and subsequent removal, of a membrane bound AMPAR subunit during LTD (Mulkey et al., 1994; Sanderson et al., 2016), which is induced by transient receptor potential cation channel subfamily V member 1 (TRPV1) receptor activation (Chávez et al., 2010). These observations collectively highlight CN as an important regulator of behavior, memory, and LTD and as a schizophrenia candidate gene that regulates *EGR3*, making this protein an integral component of our proposed biological pathway.

#### Relationship to EGR3

Evidence that CN regulates expression of *Egr3* comes from the immune system. T-cell activation induces expression of *Egr3*, which, in turn, directly regulates expression of Fas-ligand (Fasl). Expression of both *Egr3* and its target gene is blocked by the CN inhibitor cyclosporin A (Mittelstadt and Ashwell, 1998). Although a similar interaction has not been investigated in the brain, the remarkable similarity in the behavioral and electrophysiologic phenotypes of CN knockout, and *Egr3*^−/−^, mice strongly suggests this molecular pathway is conserved in the brain (Zeng et al., 2001; Miyakawa et al., 2003; Gallitano-Mendel et al., 2007). Both CN knockout and *Egr3*^−/−^ mice show deficits in LTD, spatial learning (Zeng et al., 2001; Gallitano-Mendel et al., 2007), and heightened responsiveness to handling (Gallitano-Mendel et al., 2007; supplemental data, Miyakawa et al., 2003). These intriguing initial findings support the need to determine whether this regulatory relationship between CN and *Egr3* found in the immune system is also present in the brain.

### NFATc3 (NFAT4)

The NFATs comprise a family of transcription factors that regulate activity-dependent gene expression in the immune system and the brain (Macian, 2005; Vihma et al., 2016). They reside in the cytoplasm in an inactive, phosphorylated state. Activity-triggered calcium entry into the cell stimulates CN to dephosphorylate NFAT proteins, allowing them to translocate to the nucleus where they activate expression of immediate early genes (Abdul et al., 2010). NFATs can be shuttled back into the cytoplasm via nuclear export sequences (nuclear localization signal) that, once exposed, lead to rephosphorylation of NFATs by kinases (Okamura et al., 2000) and subsequent inhibition of NFAT-mediated gene transcription (Hogan et al., 2003).

The NFAT family comprises five genes, four of which are calcium regulated and include *NFAT1-4*, and one that is osmotic tension-regulated, namely *NFAT5*, in both humans and mice (Vihma et al., 2008). NFAT proteins contain a C-terminal Rel homology domain that enables them to interact with other transcription factors (including EGRs) to co-regulate expression of downstream genes. Additional protein regions include an N-terminal domain that contains two CN binding sites (Aramburu et al., 1998; Park et al., 2000), the nuclear localization sequence, and serine residues that are sites of phosphorylation (Kiani et al., 2000).

As transcription factors, NFATs regulate expression of a wide array of genes ranging from cytokines (Klein et al., 2006), to growth factors (Hernández et al., 2001), to cell-cycle regulators (Caetano et al., 2002). In conjunction with CN, NFATs mediate a variety of physiological processes such as angiogenesis (Courtwright et al., 2009), osteogenesis (Winslow et al., 2006), and cardiovascular system development (Schulz and Yutzey, 2004). In the nervous system the NFATs have numerous roles, which include regulation of synaptic plasticity (Schwartz et al., 2009) and neurotrophin signaling (Groth et al., 2007; reviewed in Kipanyula et al., 2016).

We focus our discussion on NFATC3 (a.k.a. NFAT4), as it regulates expression of *Egr3*, and it is the only family member to show genome wide association with schizophrenia (Rengarajan et al., 2000; Schizophrenia Working Group of the Psychiatric Genomics Consortium, 2014; Pouget et al., 2016). *Nfatc3* is expressed in the adult mouse cerebellum, hippocampus, choroid plexus and ependymal cells as shown by *in situ* hybridization studies, and in the midbrain, pons, striatum, and thalamus using reverse transcription polymerase chain reaction (RT-PCR; Vihma et al., 2008). The level of expression varies across these regions with the highest expression in the mouse cerebellum and granular cell layer of the dentate gyrus in human brain tissue (Vihma et al., 2008). *In vitro* studies demonstrate that NFATC3 is one of the most highly activated NFAT members following neuronal depolarization (Vihma et al., 2016). To date, *NFATC3* remains the only NFAT family member to show genome wide association with schizophrenia and regulate gene expression of immediate early genes, both of which are discussed below.

#### Evidence for a Role in Schizophrenia

*NFATC3* maps to locus 85 of the 108 loci identified by the psychiatric genetics consortium and is, in fact, the index SNP for that locus (Schizophrenia Working Group of the Psychiatric Genomics Consortium, 2014). This initial finding has subsequently been supported by a follow-up GWAS that examined immune system-related genes from the original consortium study in an extended cohort (Pouget et al., 2016). Results of this validation study identified both *NFATC3* and *EGR1* among the six immune genes that showed genome-wide significance. Pouget et al. (2016) propose that *NFATC3*, which, like each of the 5 candidate genes they followed up, is known to have a predominantly “immune” role in peripheral organs, may be playing a non-immune role in the brain.

The vast majority of work investigating the activity and function of NFATC3 has focused on its roles in the immune system. Studies of* Nfatc3* knockout mice show reduced numbers of CD4 and CD8 cells in the thymus and spleen, and increased apoptosis of T-cells, suggesting the importance of *Nfatc3* in T-cell development and survival. No brain or nervous system phenotypes were reported in this study (Oukka et al., 1998). However emerging data suggest that NFATC3 has neuroprotective functions. It mediates astrocyte activation in response to brain damage (Serrano-Pérez et al., 2011; Yan et al., 2014), protection against apoptosis in neuronal cells *in vitro* (Butterick et al., 2010), and growth and differentiation of neuronal precursors (Serrano-Pérez et al., 2015). NFATC3 also mediates neurodegenerative effects including methamphetamine-induced apoptosis (Jayanthi et al., 2005), and apoptosis via Fas activation in lithium-induced neurotoxicity *in vivo* (Gómez-Sintes and Lucas, 2010). The latter of these is reminiscent of *Egr3*-mediated regulation of Fasl in T-cells (Mittelstadt and Ashwell, 1998, 1999; Rengarajan et al., 2000). Although there are no published studies investigating the role of NFATC3 in LTD, its position as an activity-dependent regulator of *Egr3* in this pathway leads us to hypothesize that NFATC3 will be necessary for this form of synaptic plasticity.

#### Relationship to EGR3

In the immune system, CN regulates *Egr3* expression in response to T-cell activation, an interaction that is mediated via NFATs. NFATC3 regulates expression of *Egr3* in T-cells in mice. *Egr3* expression is reduced in amount and duration in T-cells of mice lacking either NFATC2 or NFATC3, and is nearly absent in NFATC2/C3 double knockout mice. This regulation is direct, as both NFATC2 and NFATC3 are able to transactivate the* Egr3* promoter *in vitro* (Rengarajan et al., 2000). We hypothesize that, in neurons, activity-dependent activation of CN dephosphorylates NFATC3, allowing it to enter the nucleus where it binds to the *Egr3* promoter and activates its expression. While NFATC2 may also regulate neuronal expression of *Egr3*, as well as of *Egr2*, and potentially *Egr1*, the strong evidence for NFATC3 interacting with EGR3 is the basis for including this family-member as a critical component of our proposed biological pathway.

### EGR3

EGR3 is a member of the Egr family of immediate early gene zinc finger transcription factors, and is activated downstream of numerous schizophrenia candidate proteins, including NRG1, NMDAR and CN (Yamagata et al., 1994; Mittelstadt and Ashwell, 1998; Hippenmeyer et al., 2002). This family consists of four genes, *Egr1-4*, that are activated in response to a wide range of environmental stimuli, including stress (Senba and Ueyama, 1997).

*Egr3* was identified through a screen for genes homologous to *Egr1*. Like the founding family member, *Egr3* is also highly expressed throughout the brain, including in the cortex, hippocampus, basal ganglia (Yamagata et al., 1994) and suprachiasmatic nucleus (Morris et al., 1998), as well as in the immune system and other organs (Tourtellotte and Milbrandt, 1998; Mittelstadt and Ashwell, 1999; Tourtellotte et al., 2001) *Egr3* mRNA is highly induced in response to electroconvulsive seizure in hippocampal and cortical neurons, and in hippocampal dentate gyrus granule cells, by NMDAR activation (Yamagata et al., 1994), prompting further research into its role in synaptic plasticity and behavior (Gallitano-Mendel et al., 2007, 2008).

#### Evidence for a Role in Schizophrenia and LTD

Investigations published by our group revealed that *Egr3*^−/−^ mice display schizophrenia-like behavioral abnormalities, including locomotor hyperactivity that is reversed by antipsychotic treatment. In addition, these mice show immediate memory deficits, heightened reactivity to novelty, and disrupted hippocampal LTD (Gallitano-Mendel et al., 2007). Induction of LTD has recently been shown to inactivate fear-related memory *in vivo*; however, we do not know whether *Egr3* plays a role in this process (Nabavi et al., 2014). *Egr3*^−/−^ mice also display a marked resistance to the sedating effect of clozapine, and other second-generation antipsychotics, which parallels the resistance that schizophrenia patients show to the side-effects of these medications (Cutler, 2001). This phenotype may be explained, at least in part, but the reduced level of serotonin 2A receptors found in the frontal cortex of *Egr3*^−/−^ mice, a feature also seen in the brains of patients with schizophrenia (Williams et al., 2012; Selvaraj et al., 2014).

Our research also showed that *Egr3*^−/−^ mice exhibit increased aggression in response to a foreign intruder (Gallitano-Mendel et al., 2008). This response was abrogated by chronic clozapine administration, despite the fact that the *Egr3*^−/−^ mice are resistant to the sedative effects of this antipsychotic (Gallitano-Mendel et al., 2008). A similar phenomenon is seen in schizophrenia patients treated with clozapine (Hector, 1998; Chalasani et al., 2001; Chengappa et al., 2002), in whom the anti-aggressive effect of the drug can be distinguished from its sedating effect (Krakowski et al., 2006). These roles that *Egr3* plays in memory, synaptic plasticity, and behavior, and the response to antipsychotics that mimics that of patients, suggest that abnormal function of EGR3 in humans may contribute to schizophrenia pathogenesis or symptomatology.

Genetic association of *EGR3* with schizophrenia has been shown in Chinese (Ning et al., 2012; Zhang et al., 2012), Japanese (Yamada et al., 2007) and Korean (Kim et al., 2010) populations, and more recently in a population of European descent (Huentelman et al., 2015). It was also reported that decreased *EGR3* mRNA levels were observed in postmortem dorsolateral prefrontal cortex samples of schizophrenia patients compared with controls (Yamada et al., 2007). In addition, *EGR3* was identified in a screen for genes that are expressed at reduced levels in the brains of schizophrenia patients that do not smoke tobacco, and are normalized to control levels in patients that smoke. Notably, *EGR3* levels followed a pattern identical to that of *CN* in the study, consistent with its regulation by CN in the proposed biological pathway (Mexal et al., 2005).

The 8p chromosomal region, where *EGR3* resides, is a long-recognized hub for schizophrenia associations (Suarez et al., 2006; Lohoff et al., 2008). A recent review highlighted that *EGR3* was among 20 genes of interest for schizophrenia, map to the 8p locus (Tabarés-Seisdedos and Rubenstein, 2009). Association of an *EGR3* SNP with decreased prefrontal hemodynamic response was observed during a verbal fluency task in both healthy controls and schizophrenia patients (Nishimura et al., 2014).

Both human and *in vivo* animal studies suggest that overexpression of EGR3 may also negatively impact non-neural physiological processes, including immune system function. High levels of *EGR3* positively correlate with levels of proinflammatory cytokines in peripheral monocytes of schizophrenia patients (Drexhage et al., 2010). This may be mediated through the Triggering receptor expressed on myeloid cells 1 (TREM-1), a key regulator of inflammation in both brain microglia and peripheral monocytes, as EGR3 directly binds the TREM-1 promoter in monocytes (Weigelt et al., 2011).

These data support the hypothesis that EGR3 may function as a master regulator of multiple processes that are relevant to the pathophysiology of schizophrenia. In line with this theory, bioinformatic analyses have predicted EGR3 to be a central modulator of a regulatory network of microRNAs and transcription factors associated with schizophrenia (Guo et al., 2010). Despite these findings, *EGR3* was not identified as a gene residing at one of the 108 loci associated with schizophrenia. Genome-wide association studies from the PGC schizophrenia studies suggest that none of the SNPs in the region including *EGR3* showed a significant association. The SNP that showed the lowest *p* value was an intergenic SNP, rs12541654 (*p* = 0.23, OR = 0.99)^1^. However other EGR family members, such as* EGR1*, and EGR coregulatory binding factor NGFI-A Binding Protein 2 (*NAB2*), are each index SNPs at one of the 108 loci (Schizophrenia Working Group of the Psychiatric Genomics Consortium, 2014). EGR3 binds to NAB2 to co-regulate expression of target genes, and is involved in co-regulatory feedback relationships with EGR1, as well EGR1, EGR2 and EGR3 can regulate *NAB2* expression, demonstrating a functional interaction of both of these schizophrenia associated genes in the proposed pathway (Svaren et al., 1996; Mechta-Grigoriou et al., 2000; Kumbrink et al., 2005, 2010; Srinivasan et al., 2007).

### ARC

One of the first EGR3-target genes to be elucidated was *Arc* (Li et al., 2005). *Arc* was originally identified as a novel transcript in the adult rat brain that was rapidly and strongly induced in response to electroconvulsive seizures (Link et al., 1995; Lyford et al., 1995). Induction of *Arc* was observed *in vitro* in PC12 cells following exposure to growth factors such as nerve growth factor (NGF) and epidermal growth factor (EGF; Lyford et al., 1995). *Arc* mRNA and protein are enriched in neuronal dendrites, and ARC protein colocalizes with the actin cytoskeletal matrix (F-actin; Lyford et al., 1995). These observations led to its name “ARC”. Further characterization of the role of ARC protein in cytoskeletal function revealed that it also associates with microtubules and microtubule associated protein (MAP2; Fujimoto et al., 2004). ARC was also shown to maintain phosphorylation of cofilin, the actin depolymerization factor, and to promote F-actin formation (Messaoudi et al., 2007).

ARC is capable of localizing to NMDAR and postsynaptic density (PSD) 95 protein complexes (Husi et al., 2000; Donai et al., 2003) suggesting that it may be involved in synaptic neurotransmission. ARC also binds to multiple components of the clathrin-dependent endocytic machinery including endophilin and dynamin, and decreases total and surface AMPAR (GluR1) protein levels via endocytosis in hippocampal neurons *in vitro* (Chowdhury et al., 2006). Numerous proteins bind to ARC to mediate its many important roles in the nervous system. Notably 4–8 of these binding partners, termed “ARC complex proteins”, were identified in schizophrenia GWAS (Kirov et al., 2012; Fromer et al., 2014; Purcell et al., 2014) and are discussed in depth in the next section.

#### Evidence for a Role in Schizophrenia

The behavioral phenotype of *Arc*^−/−^ mice was recently characterized and revealed schizophrenia-like abnormalities. These include deficits in prepulse inhibition and recency discrimination (indicative of cognitive dysfunction), impairments in response to social novelty, hyperactivity in response to amphetamine administration, and region-specific alterations in dopamine (Managò et al., 2016). Though they retain the ability to form intact short-term memory, *Arc*^−/−^ mice fail to form long lasting memories.

The necessity of *Arc* for normal memory and behavior in mice suggest that dysfunction of *ARC* in humans could result in abnormalities that increase risk to develop psychiatric illness. Indeed, *ARC* mRNA expression is decreased in the frontal cortex of schizophrenia patients (Guillozet-Bongaarts et al., 2014). In addition, genes encoding proteins that bind to ARC, “ARC-complex proteins”, were identified in several large-scale, genome-wide schizophrenia genetic association studies. Kirov et al. (2012) reported that *de novo* CNVs in the genes *DLG1*, *DLG2*, *DLGAP1*, *CYFIP1*, each of which encode ARC-complex proteins, show significant enrichment in individuals with schizophrenia. A separate case-control study showed an enrichment of disruptive mutations in genes encoding ARC-complex proteins in schizophrenia cases vs. controls (Purcell et al., 2014). The nine mutations identified in this study included nonsense, essential splice site, and framseshift mutations, that occurred in nine genes, and were found *only* in schizophrenia cases, with none occurring in controls (Purcell et al., 2014). In addition, the study by Fromer et al. (2014) that identified increased *de novo* mutations in NMDAR-complex gene in schizophrenia patients also revealed that these mutations are enriched in genes encoding proteins associated with the ARC-complex. Fromer et al. (2014) showed that loss of function mutations are 17-fold enriched in genes encoding ARC-complex proteins in their cohort and 19-fold enriched in the data set from the Purcell et al. (2014) study. They concluded that “ARC disruption has particularly strong effects on disease risk” (Fromer et al., 2014).

These and other findings implicated ARC as a critical protein in a synaptic pathway involving voltage gated calcium channels, NMDARs, PSD95, FMRP, mGLuR and AMPARs, in schizophrenia risk (Hall et al., 2015). Although these studies did not report genetic association of variations in the *ARC* gene, itself, with schizophrenia, our group subsequently reported the first association of a SNP in the *ARC* gene with schizophrenia in two separate populations (discussed below; Huentelman et al., 2015). More recently, hypermethylation of eight CpG sites, and presence of several rare variants that reduce reporter gene activity, were reported in the putative ARC promoter region of schizophrenia patients vs. controls (Chuang et al., 2016). These findings indicate that altered genetic and epigenetic regulation of *ARC* expression, specifically that reduces ARC function, may increase risk for schizophrenia.

#### Role in LTD

Like many of the upstream proteins in the proposed pathway, ARC is also required for hippocampal LTD. NMDA-mediated LTD deficits in Schaffer collateral-CA1 synapses were found in slices from *Arc*^−/−^ mice compared to wildtype mice (Plath et al., 2006). In addition, mGLuR-mediated LTD was shown to depend on rapid translation of ARC, and ARC-mediated AMPAR endocytosis, in hippocampal neurons (Waung et al., 2008).

Neuronal activity stimulates expression of ARC, which then accumulates at the spines of *inactive* synapses, effectively “tagging” these synapses for remodeling. This process is dependent on Ca^2+^/calmodulin-dependent protein kinase II (CamKIIβ), and results in ARC-mediated AMPA receptor endocytosis (Okuno et al., 2012). Minatohara et al. (2016) suggest that ARC expression and localization during LTD may increase the strength of the active synapses by causing endocytosis of AMPARs at the inactive synapses. *Arc*^−/−^ mice also show decreased spine density and increased spine width in CA1 pyramidal neurons and DG cells (Peebles et al., 2010), suggesting that ARC plays a critical role in maintenance of spine morphology that may impact learning and behavior. These data suggest that decreased *ARC* expression results in deficits in LTD.

#### Relationship to EGR3

Previous research has shown that *Arc* is a transcriptional target of EGR3 (Li et al., 2005). Our proposed biological pathway (Gallitano-Mendel et al., 2007, 2008) combines environment stress-responsive proteins *EGR3*, CN, and NMDARs, each of which are associated with schizophrenia susceptibility. Since mice deficient for these proteins share phenotypes of LTD and memory deficits, similar to mice lacking *Arc*, we hypothesized that the *ARC* gene should likewise be a schizophrenia candidate gene.

To test this hypothesis, we used next generation sequencing to resequence the *ARC* gene, and flanking regions, from schizophrenia patient and control subjects from two separate ethnic groups. These studies revealed association between the *ARC* SNP and schizophrenia in both ethnic groups (Huentelman et al., 2015). In a separate study, we found that the *ARC* SNP was associated with response to cognitive remediation therapy in a cohort of new onset psychosis patients (Breitborde et al., 2017). Recently, a heritable chromosomal microdeletion that encompasses several genes, including *ARC*, was also shown to be associated with several neurodevelopmental psychiatric disorders such as attention deficit hyperactivity disorder and autism spectrum disorder (Hu et al., 2015) in addition to schizophrenia. Overall, substantial evidence suggests that ARC, an important regulator of synaptic function, memory, and LTD, influences risk for schizophrenia, and possibly other neuropsychiatric illnesses. As a direct target of EGR3, *ARC* represents an important output element of our proposed biological pathway.

## Additional Genes That Interact with the Pathway

### Neuregulin-1

*NRG1* was one of the first schizophrenia candidate genes to be identified using molecular genetic methods at a genomic locus defined by linkage analysis studies in family pedigrees (Stefansson et al., 2002). It has subsequently been validated in numerous populations, and supported by preclinical studies (recently reviewed in, Mostaid et al., 2016). NRG1 regulates *EGR3* in human muscle and breast cancer cell lines (Sweeney et al., 2001; Jacobson et al., 2004). In mice NRG1 regulates *Egr3* expression in developing muscle cells (Jacobson et al., 2004), and this regulation is required to maintain development of muscle spindles. These studies demonstrate a functional link between these two schizophrenia-associated genes in two types of human cell lines, and *in vivo* in mice (Hippenmeyer et al., 2002; Jacobson et al., 2004; Herndon et al., 2013). Although this regulatory relationship has not yet been validated in the brain, we hypothesize that a similar regulatory relationship may functionally link these two schizophrenia-associated genes in the central nervous system as well.

No studies investigating LTD in NRG1 deficient mice have been reported. However, the fact that NRG1 regulation of *Egr3* in the muscle spindle is mediated by SRF (Jacobson et al., 2004; Herndon et al., 2013), and loss of either *Srf* or *Egr3* results in memory and LTD deficits (Etkin et al., 2006; Gallitano-Mendel et al., 2007), leads us to predict that NRG1 should also play a critical role in this form of hippocampal synaptic plasticity. Indeed, NRG1 impairs endocannabinoid 2-arachidonoylglycerol (2-AG)-mediated LTD in rat hippocampal slices (Du et al., 2013).

### Serum Response Factor (SRF)

We have previously highlighted SRF as a protein in the proposed biological pathway for schizophrenia susceptibility based on its requirement for both novelty memory and LTD in mice (Etkin et al., 2006), as well its regulatory interactions with other proteins in the pathway (Etkin et al., 2006; Gallitano-Mendel et al., 2007). SRF is activated downstream of NRG1 in mouse muscle cells (Herndon et al., 2013, 2014). *In vitro* studies demonstrate that NRG1 is capable of stimulating *SRF* expression in human HeLa cells expressing the NRG1 receptor ErbB4, and that this is mediated by mitogen-activated protein kinase (MAPK; Eto et al., 2010). SRF is also activated by CN/NFAT, in combination with other factors, in lymphocytes (Lin et al., 2002; Hao et al., 2003). In turn, SRF regulates expression of *Egr1* and *Egr2* in the brain in response to novelty, and mediates the regulation of *Egr3* by NRG1 in muscle spindle development (Hao et al., 2003; Ramanan et al., 2005; Etkin et al., 2006; Herndon et al., 2013). SRF has also been shown to regulate *Egr3* expression in the hippocampus (personal communication from Naren Ramanan).

Together with another transcription factor Elk-1, SRF regulates expression of *Egr1* in response to chemically-induced LTD (by 3,5-dihydroxyphenylglycine, DHPG; Lindecke et al., 2006). And *Srf*-deficient mice have deficits in LTD (Etkin et al., 2006). Interestingly two *NRG1* schizophrenia-associated SNPs from the original Icelandic haplotype occur in regions that show predicted binding sites for SRF that are abolished by presence of the SNPs (Law et al., 2006). Although we are unaware of studies demonstrating that genetic polymorphisms in *SRF* are directly associated with risk for schizophrenia at this time, we hypothesize that the positioning of SRF in this pathway, and its requirement for hippocampal LTD, will lead to identification of such roles in the future.

### EGR1

*Egr1* is the founding member of the Egr family of immediate early gene transcription factors. It was identified as a gene activated in response to application of NGF to PC-12 cells in culture, and thus named “NGF inducible A” NGFI-A (O’Donovan et al., 1999). *Egr1* was independently identified by other laboratories, which accounts for its numerous aliases (zif-268, Krox-24, NGFI-A, TIS8, ZIF-268, ZNF225). *Egr1* is required for long-term memory formation and late-phase LTP (Cole et al., 1989; Worley et al., 1993).

While a number of studies have identified associations between* EGR1* and schizophrenia, the most significant is that of the 2014 Schizophrenia Working Group of the Psychiatric Genomics Consortium GWAS, in which *EGR1* resides in region 69 of the 108 loci associated with the illness, and contains the Index SNP for that region (Schizophrenia Working Group of the Psychiatric Genomics Consortium, 2014). In a follow-up study examining the consortium dataset, Pouget et al. (2016) identified six independent regions from the 108 loci where the SNP with the lowest association *p*-value (the Index SNP) was in an immune gene. *EGR1* was one of the six.

Postmortem brain tissue studies have also identified* EGR1* as one of a small number of genes differentially expressed in schizophrenia patients compared with controls (Pérez-Santiago et al., 2012). Studies have also identified differences in mRNA levels of *EGR1* in peripheral blood cells (Cattane et al., 2015; Xu et al., 2016; Liu et al., 2017), and one study in fibroblasts (Cattane et al., 2015), of schizophrenia patients compared with controls Interestingly, the differences in *EGR1* mRNA levels identified by the two research groups were in opposite directions, which may be due to methodological differences. In particular, the two studies reporting reduced peripheral blood cell levels of *EGR1* were examining schizophrenia patients during an acute psychotic episode, while the other study did not specify the patients’ current symptom status (Cattane et al., 2015; Xu et al., 2016; Liu et al., 2017).

Studies in mice deficient for *Egr1* demonstrate that both long-term memory and LTP require function of this immediate early gene (Jones et al., 2001; Bozon et al., 2003). Furthermore, over-expression of* Egr1* facilitates hippocampal LTP and enhances long-term memory of spatial location (Penke et al., 2013). Although *Egr1* is upregulated in response to LTD induction by DHPG (Lindecke et al., 2006), loss of *Egr1* has not been reported to result in LTD deficits.

As the founding member of the EGR family, EGR1 shares gene sequence homology, and DNA binding element recognition, with EGR3. EGR1 and EGR3 are activated by many of the same stimuli and are expressed in the same cells in the brain (Yamagata et al., 1994), though their proteins follow different temporal patterns of expression and perdurance (O’Donovan et al., 1998).

Together EGR1 and EGR3 bind to the promoter, and induce expression of, their coregulatory factor *NAB2* in cells of neuroectodermal origin (Kumbrink et al., 2010). NAB2 protein, via interaction with the EGRs, subsequently feedback inhibits its own expression (Kumbrink et al., 2010). These regulatory relationships that EGR1 shares with EGR3 and other proteins in the proposed pathway, as well as with *NAB2*, another GWAS-implicated gene, strengthen the likelihood that the position of *EGR1* as an Index SNP in the 108 loci indicates an actual role for EGR1 in schizophrenia susceptibility.

### NGFI-A Binding Protein 2 (NAB2)

NAB2 is an activity-dependent immediate early gene that functions as a transcriptional coregulatory protein by binding to a specific recognition domain on EGR family transcription factors EGR1, EGR2 and EGR3. In cells of neuroectodermal origin EGR1 and EGR3 bind in concert to the promoter of the *NAB2* gene to induce its expression (Kumbrink et al., 2010). NAB2 protein has both co-activation and co-repression actions. Once bound to the EGRs, NAB2 feedback inhibits its own expression, as well as that of EGRs (Svaren et al., 1996; Mechta-Grigoriou et al., 2000; Kumbrink et al., 2010).

The regulatory relationships with schizophrenia-associated EGRs (EGR1 and EGR3) suggest a role for NAB2 in neuropsychiatric illness. Direct support for possible association between *NAB2* and schizophrenia comes from the 2014 Schizophrenia Working Group of the Psychiatric Genomics Consortium GWAS. *NAB2* is located at region 20 of the 108 loci, and is one of the genes at the site of the locus’ Index SNP (Schizophrenia Working Group of the Psychiatric Genomics Consortium, 2014).

The majority of studies involving NAB2 have investigated its role in the immune system and cancer biology (Collins et al., 2006, 2008; Hastings et al., 2017). The role of NAB2 in the nervous system is much less well investigated, and the functions of NAB2 in the brain are still largely unknown. As an immediate early gene, *Nab2* expression is induced in the brain in response to stimuli (Jouvert et al., 2002). Among the stimuli that activate *Nab2* expression in neurons is BDNF (Chandwani et al., 2013). In the peripheral nervous system loss of both *Nab2*, and its family member *Nab1*, results in severe myelination defects (Le et al., 2005).

No studies have yet been published examining the role of NAB2 in memory or hippocampal synaptic plasticity, or in behavior. However, unpublished data from our laboratory indicate significant behavioral abnormalities in mice lacking only *Nab2* (Gallitano, unpublished observation). Despite the paucity of published work on the role of NAB2 in the brain, the regulatory relationships between NAB2, EGR1 and EGR3 link this gene not only to other specific schizophrenia-associated genes, but also to the proposed pathway for illness association.

### Brain-Derived Neurotrophic Factor (BDNF)

Numerous studies have associated BDNF with mental illnesses, including schizophrenia (Islam et al., 2017). The requirement for BDNF, particularly its precursor (pro) form, for synaptic plasticity and memory, suggests a mechanism linking BDNF to neuropsychiatric disorders (reviewed in, Carlino et al., 2013). Specifically, the human *BDNF* polymorphism that converts the 66 position amino acid valine to a methionine (Val66Met) has been associated not only with mental illness risk, but also with memory (Egan et al., 2003; Hariri et al., 2003; Hashimoto et al., 2008; van Wingen et al., 2010).

BDNF is included in the proposed pathway as it has been shown to induce expression of *Egr3* in the mouse brain as an essential step in regulating expression of GABAA receptor alpha 4 subunit (Roberts et al., 2006; Kim et al., 2012). In a separate article submitted to this Research Topic issue (Meyers et al., 2017a), we demonstrate that *Egr3* is required for expression of *Bdnf* in the mouse dentate gyrus 1 h following seizure, a stimulus that induces high level *Bdnf* expression in this hippocampal region. Thus, BDNF may function both upstream, as well as downstream, of *Egr3*.

It was recently reported that BDNF-deficient mice display defects in hippocampal LTD (Novkovic et al., 2015; Bukalo et al., 2016). In addition, exogenous application of the precursor (pro) peptide of BDNF facilitates development of LTD, a process that requires function of GLUN2B containing NMDARs (Mizui et al., 2015; Kojima and Mizui, 2017). Notably, the hippocampal Schaffer collateral neurons in *Egr3*^−/−^ mice, that fail to develop LTD, are unresponsive to the GLUN2B selective antagonist ifenprodil (Gallitano-Mendel et al., 2007), suggesting that GLUN2B-containing NMDARs are critical for both BDNF- and *Egr3*-, mediated LTD. These findings support a position for BDNF in our proposed pathway for neuropsychiatric illness risk.

## Discussion

In this article, we review evidence supporting an activity-dependent biological pathway that incorporates numerous schizophrenia candidate genes with critical roles in the regulation of memory and LTD, and that culminates in activation of the immediate early gene transcription factor *EGR3*. The unique position of immediate early gene transcription factors, at the nexus between environmental events and regulation of the neuronal response to activity, makes them ideally suited to account for both the genetic and environmental contributions to schizophrenia.

Additional proteins implicated in risk for schizophrenia, but that have not yet been reported to affect LTD, interact with this pathway either as upstream activators (e.g., NRG1) or as transcriptional regulators (e.g., EGR1 and NAB2). Individual genes in this pathway mediate neuroprotective functions including myelination, vascularization, growth factor regulation, and synapse formation, abnormalities in which are found in schizophrenia (Milbrandt, 1987; Mechtcheriakova et al., 1999; Jones et al., 2001; Jessen and Mirsky, 2002; Fahmy et al., 2003; Fahmy and Khachigian, 2007; Gallitano-Mendel et al., 2007). Dysfunction in any of the genes of this pathway would result in disruption of these processes, and may thereby account for the neuropathologic and clinical features of schizophrenia, including deficiencies white matter, brain volumes, and cerebral blood flow, reduced synaptic spine density, and deficits in memory and cognitive processing (Ingvar and Franzen, 1974; Saykin et al., 1991; Glantz and Lewis, 2000; Moises et al., 2002; Davis et al., 2003; Nabavi et al., 2014).

Moreover, numerous genes in the pathway have critical functions in the immune system, particularly in T-cell activation, underscoring a longstanding recognition of a relationship between immune system and risk for schizophrenia. Finally, numerous genes that are part of the originally-defined pathway, as well as others that interact with proteins in the pathway, have subsequently been identified in the 108 loci GWAS (Schizophrenia Working Group of the Psychiatric Genomics Consortium, 2014). Notably, each of the pathway genes that is at one of the 108 schizophrenia loci is, in fact, *the* Index SNP at that locus. Together these findings strongly support the biological relevance of our proposed pathway in schizophrenia.

In the “Introduction” section, we highlighted that there are two unanswered questions at the forefront of psychiatric genetics: (1) how can so many genes contribute susceptibility to schizophrenia? and (2) how do genes implicated in risk for schizophrenia interact with environmental factors to give rise to the disorder? The concept of a biological pathway addresses the first unanswered question by hypothesizing that numerous genes share roles in a much smaller number of critical functional processes. Disruption in these key biological processes create risk for schizophrenia.

**Figure 2:**
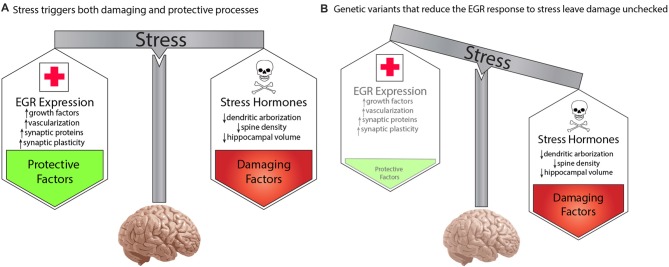
A developmental model for genetic predisposition to stress susceptibility.** (A)** Stress hormones cause detrimental effects on the brain, such as decreased dendritic arborization, reduced density of synaptic spines, and decreased regional brain volumes, which are seen in schizophrenia. Stress also activates EGR family immediate early genes, which mediate numerous beneficial processes, such as growth factor response, vascularization, synaptic protein expression, and synaptic plasticity. The balance between these processes protects the brain from damage under stressful conditions. **(B)** Individuals carrying genetic variants that impair the activation of pathway proteins may be fine under normal conditions. However, in the context of stress, the damaging effects override the insufficient protective response, resulting in neurotoxic insults which, if sustained or repeated, may result in the neuropathology that gives rise to symptoms of schizophrenia. In this manner, genetic variations in genes of the proposed pathway create a predisposition to develop schizophrenia in a manner dependent upon the stress history of an individual.

To account for the effect of environment, we propose a model whereby genetic variations that decreased function of any protein in the pathway will result in increased risk for schizophrenia in a manner that is dependent upon the stress history of an individual. Stress is commonly thought of as detrimental. Specific negative consequences of stress demonstrated in animal and human studies include stress-hormone mediated decreases in dendritic arborization and spine density and reduced regional brain volumes (reviewed in Lupien et al., 2009). However, despite the pervasive experience of stress throughout life, most people do not develop severe mental illness. This is presumably because stress also activates molecular and cellular processes that are protective. The balance of damaging and protective responses to stress creates resilience and maintain homeostasis. This is shown in Figure 2A.

We propose that the activity-dependent biological pathway we have described here represents a component of the healthy biological response to stress. The normal activation of the pathway in response to stress triggers molecular and cellular processes that protects the brain from the harmful effects of stress hormones, and other detrimental elements of the stress response. While extreme, or unremitting, stress conditions may over-ride the buffering abilities of this protective arm of the stress response, it is sufficient to withstand typical episodic stress that occurs in daily life.

However, if an individual carries a genetic variation that decreases the responsiveness of a critical protein in our proposed pathway (Figure 2B), then exposure to stress will result in the detrimental effects of stress that are not sufficiently balanced by the protective elements. This preponderance of damaging effects over time is hypothesized to cause the neuropathologic features of schizophrenia that presumably give rise to symptoms of the illness. In the absence of sufficient stress, however, the lower level of function of the EGR pathway may be sufficient to prevent neuropathological consequences. This can explain how two individuals may carry the same genetic variation, but be discordant for schizophrenia.

In this manner, the proposed model would account for the dual genetic and environmental contributions to schizophrenia susceptibility, a crucial feature that has not been accounted for by previous gene or pathway models. We hope that this pathway and model provide the structure for investigation of additional components that may contribute to the neuropathology underlying schizophrenia and other neuropsychiatric disorders, advancing the field toward identification of more effective therapies, and perhaps one day a cure, for these illnesses.

We have focused on the role of our proposed pathway in schizophrenia due to the large body of supporting studies from human genetic, post-mortem, and basic science animal studies. However, we hypothesize that this pathway is relevant for other neuropsychiatric and neurodegenerative disorders in which stress plays a role and memory is affected. Indeed, *EGR* genes, and *EGR3* in particular, has been implicated in bipolar disorder, depression and Alzheimer’s disease (Hokama et al., 2014; Pfaffenseller et al., 2016; Francis et al., 2017). Although in-depth discussion of these disorders is beyond the scope of this review, we look forward to application of our proposed pathway to these illnesses by others in the future.

## Author Contributions

ALG conceived the hypothesis that is the focus of the manuscript. KKM and ALG were both responsible for collecting literature, review and writing of the manuscript.

## Conflict of Interest Statement

The authors declare that the research was conducted in the absence of any commercial or financial relationships that could be construed as a potential conflict of interest.

## Footnotes

^1^http://zzz.bwh.harvard.edu/code.html

## Supplementary Material

TABLE S1 Supplemental table contains details regarding human variant studies discussed in the review. For each study discussed the associated gene, protein, variant, *p* value, effect size (if reported), and major findings have been summarized.
